# Out-of-hospital cardiac arrest: A data-driven visualization of collaboration, frontier identification, and future trends

**DOI:** 10.1097/MD.0000000000034783

**Published:** 2023-08-18

**Authors:** Yue Li, Zhaoying Li, Chunjie Li, Wei Cai, Tao Liu, Ji Li, Haojun Fan, Chunxia Cao

**Affiliations:** a College of Management and Economics, Tianjin University, Tianjin, China; b Chest hospital, Tianjin University, Tianjin, China; c Department of Prevention and Therapy of Cardiovascular Diseases in Alpine Environment of Plateau, Characteristic Medical Center of the Chinese People’s Armed Police Forces, Tianjin, China; d Institute of Disaster and Emergency Medicine, Tianjin University, Tianjin, China; e Wenzhou Safety (Emergency) Institute, Tianjin University, Wenzhou, China.

**Keywords:** bibliometric analysis, emergency medical services, epidemiology, extracorporeal cardiopulmonary resuscitation, out-of-hospital cardiac arrest

## Abstract

One of the main causes of death is out-of-hospital cardiac arrest (OHCA), which has a poor prognosis and poor neurological outcomes. This phenomenon has attracted increasing attention. However, there is still no published bibliometric analysis of OHCA. This bibliometric analysis of publications on OHCA aimed to visualize the current status of research, determine the frontiers of research, and identify future trends. Publications on OHCA were downloaded from the web of science database. The data elements included year, countries/territories, institutions, authors, journals, research areas, citations of publications, etc. Joinpoint regression and exponential models were used to identify and predict the trend of publications, respectively. Knowledge domain maps were applied to conduct contribution and collaboration, cooccurrence, cocitation, and coupled analyses. Timeline and burst detection analysis were used to identify the frontiers in the field. A total of 3 219 publications on OHCA were found from 1998 to 2022 (average annual percentage change = 16.7; 95% CI 14.4, 19.1). It was estimated that 859 articles and reviews would be published in 2025. The following research hotpots were identified: statement, epidemiology, clinical care, factors influencing prognosis and emergency medical services. The research frontier identification revealed that 7 categories were classified, including therapeutic hypothermia, emergency medical services, airway management, myocardial infarction, extracorporeal cardiopulmonary resuscitation, stroke foundation and trial. The burst detection analysis revealed that percutaneous coronary intervention, neurologic outcome, COVID-19 and extracorporeal cardiopulmonary resuscitation are issues that should be given continual attention in the future. This bibliometric analysis may reflect the current status and future frontiers of OHCA research.

## 1. Introduction

Out-of-hospital cardiac arrest (OHCA) is a leading cause of mortality worldwide,^[1]^ accounting for millions of deaths.^[2,3]^ The incidence rates of OHCA are 52.5 per 100,000 person-years in Asia, 86.4 per 100,000 person-years in Europe, 98.1 per 100,000 person-years in North America, and 112.9 per 100,000 person-years in Australia.^[3]^ Research on OHCA has attracted an increasing amount of attention.^[4]^ Aung Myat, Marcus Eng Hock Ong and Christian Hassager explored current concepts, prehospital management and in-hospital intervention strategies of OHCA, respectively.^[1,5,6]^ Thomas Rea used the Utstein formula and the clinical framework of the links in the chain of OHCA survival to reflect on historical developments, current conditions, and potential future outcomes.^[7]^ Faruk Daniş analyzed scientific articles on cardiopulmonary resuscitation (CPR) by statistical methods.^[8]^ Juliette Thibodeau described and summarized the body of literature related to OHCA in Africa using a scoping review.^[9]^

It is challenging for traditional review papers to efficiently examine and condense a lengthy period of time and a large number of publications on a certain topic.^[10]^ Researchers can conduct macroscopic and microscopic analyses of a large number of publications using a quantitative analysis technique known as “bibliometric analysis,” which is based on mathematical statistics. This allows researchers to investigate the status and development trends of a particular research field.^[11–13]^ To our knowledge, no comprehensive bibliometric analysis on OHCA has been performed. Therefore, reviewing all pertinent publications is important to close this research gap and advance the theoretical framework in the field of OHCA as a result of the strengthening of the theoretical knowledge base and the development of the new knowledge frontier.

In the present study, we aimed to perform a bibliometric analysis of publications on OHCA in order to visualize the current status of this field and to identify research frontiers and future trends.

## 2. Methods

### 2.1. Data source and retrieval

We searched the web of science (WoS) core collection database (by Clarivate Analytics) between 1998 and 2022 for this study. The information was obtained from a consultation carried out on September 24, 2022. The study titles were scanned to omit papers that were not directly connected to the topic.^[8]^ Based on a preliminary review, the query equation for the advanced search was as follows: TI = (“Out-of-hospital cardiac arrest” OR “pre-hospital cardiac-arrest” OR “out-hospital cardiac arrest” OR “out of hospital cardiac arrest” OR “out of hospital heart arrest” OR “out-of-hospital heart arrest”). We used the Boolean operator “or” to ensure that at least 1 search term existed to find exact expressions. Literature types included review papers and research articles. The data elements obtained from the WoS core collection database in this study were as follows: year, countries/territories, institutions, authors, journals, research areas, citations of publications, etc.

### 2.2. Statistical analysis

This study preprocessed all data with co-occurrence (COOC) 12.8, including removing duplicate documents, filling missing data, merging synonyms and deleting meaningless words. When filling missing data, considering the lack of keywords given by the authors, this paper chose the given keywords plus as the supplement of keywords given by the authors to improve the accuracy of this study.

Joinpoint regression was used to identify annual trends with significant changes and the turning point in the number of global publications by calculating the annual percentage change and average annual percentage change using the Joinpoint Regression Program, version 4.9.0.0 (National Cancer Institute). An exponential model was applied to predict the number of publications in the coming years using Microsoft Excel 2021. The *R^2^* value was used to evaluate the compatibility of the exponential model with the data and model success.

The data elements of publications were analyzed using tabulation and visual drawing. Additionally, knowledge domain maps for the contribution and collaboration of authors, institutions, countries/territories and journals, cooccurrence of keywords, cocitation, and coupled analysis were plotted using VOSviewer 1.6.18. Impact factors (IF) of journals were based on the latest Journal Citation Reports 2022. A keyword timeline view was plotted using CiteSpace 6.1.R3.

**Figure 1.**
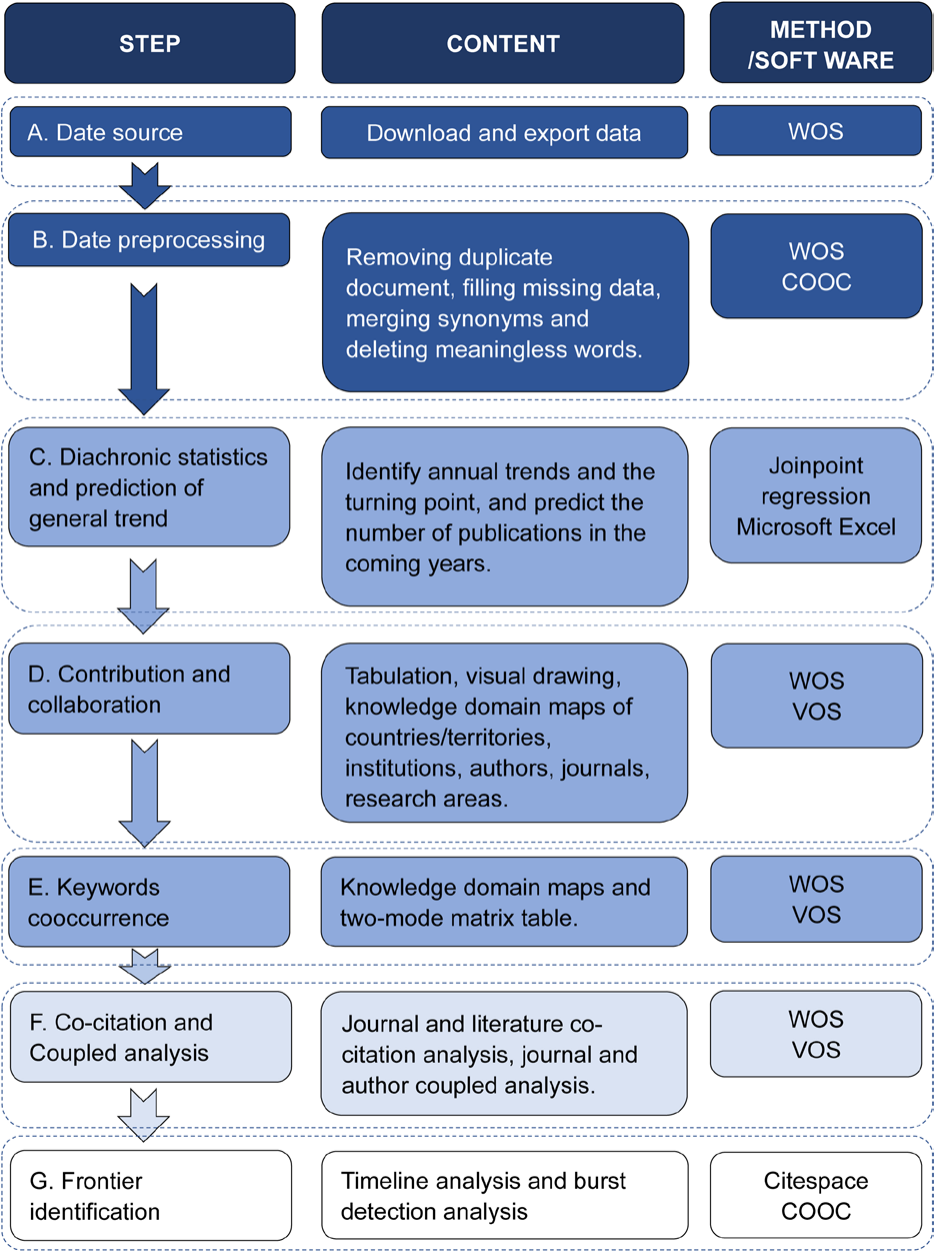
The framework of bibliometric analysis methods.

In this study, COOC software was used to construct the 2-mode matrix, conduct hierarchical cluster analysis of high-frequency keywords and journals, and identify keywords with the strongest citation bursts. Ward minimum-variance method was used to cluster the data in the 2-mode matrix clustering study, and the distance between the samples was calculated using the Euclidean distance algorithm. The construction of the 2-mode matrix relation and the detailed algorithm process are presented in the Supplementary materials (see Supplementary materials 1, http://links.lww.com/MD/J562, Supplemental Digital Content, which illustrates the construction of the 2-mode matrix). The framework for the software and analysis techniques used in this work is shown in Figure 1.

## 3. Results and discussion

### 3.1. Diachronic statistics and prediction of general trend

**Figure 2.**
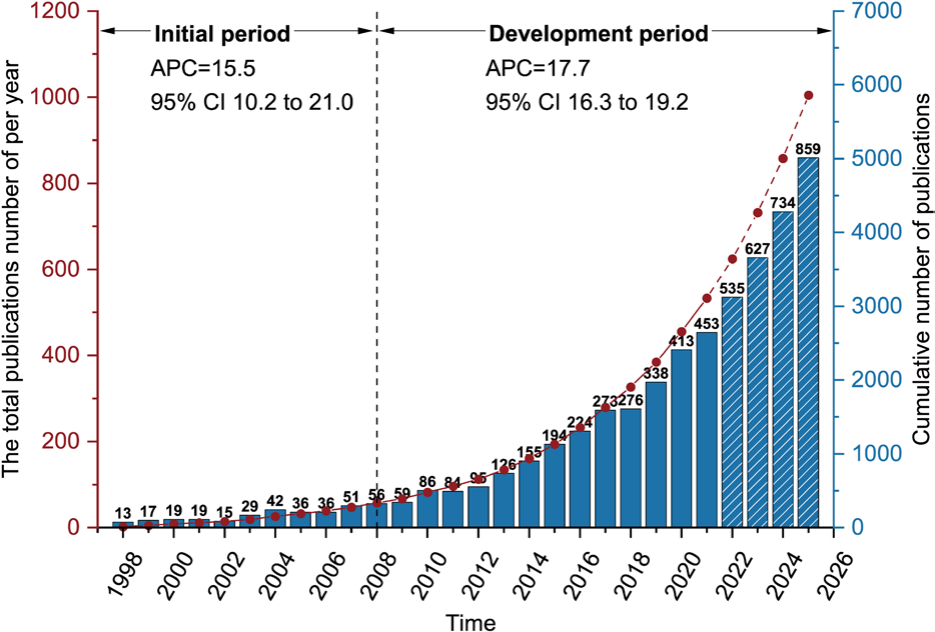
Diachronic statistics and estimation of the number of articles and reviews.

Notes: The dashed and shaded bars represented the estimation of the number of articles per yr and cumulative number of articles and reviews, respectively.

A total of 3219 publications on OHCA were found in the WoS core collection database (average annual percentage change = 16.7; 95% CI 14.4, 19.1). A total of 93.66% (n = 3015) were articles, and 6.34% (n = 204) were reviews. The yearly quantitative distribution of OHCA publications shows the field maturity as well as its state of development and knowledge acquisition. A vast increase in the number of publications was observed over this period, with annual publications growing from 13 in 1998 to 453 in 2021 and an annual growth rate of 16.7 (95% CI 14.4, 19.1) (Fig. 2). This study found that the first article was published in 1998. Thompson RJ predicted neurologic outcomes and mortality by logistic regression models based on the hospital records of 127 cardiac arrest patients in the first article.^[14]^

According to the number of articles and reviews, this period was preliminarily divided into 2 stages. Stage 1, from 1998 to 2008, was considered the initial period when the average number of papers per year was 30, with an annual growth rate of 15.5 (95% CI 10.2, 21.0). In this initial period, only a few researchers had worked on OHCA, but there was also a substantial amount of high-quality literature in this field. For example, Bernard SA compared the outcome of moderate induced hypothermia with normothermia in comatose survivors of OHCA based on a prospective, controlled trial.^[15]^ Stage 2, since 2008, was known as the development period when the number of publications increased sharply, with an annual growth rate of 17.7 (95% CI 16.3, 19.2), indicating that people had paid more attention to OHCA. During this period, annual publication growth accelerated. The papers that were published displayed a varied growth tendency, and their research content started to increase. In 2013, the number of articles and reviews exceeded 100 for the first time. To date, 306 articles and reviews have been published as of September 24, 2022. Research interest in areas that are relevant to OHCA is still expanding quickly, indicating that OHCA is widely used and has a significant impact on the global scientific community.

The number per year obtained using the exponential model is shown in Figure 1 with a dashed line to estimate the number of articles and reviews that may be published in 2022 and beyond. According to the results of the exponential model, it was estimated that 535 articles would be published in 2022 and 859 articles would be published in 2025 (*R^2^* = 0.98), and the number of articles will continue to rise.

### 3.2. Contribution and collaboration

#### 3.2.1. Active countries.

The distribution of the number of articles by country is shown in Table S1 to identify the productive of countries/territories (see Table S1, http://links.lww.com/MD/J563, Supplemental Digital Content, which illustrates the number of publications on out-of-hospital cardiac arrest of countries/territories, institutions, authors, journals, research areas). There were as many as 75 countries/territories involved in this field. The 3 countries with the most articles published were the United States of America (USA) (824, 25.60%), Japan (424, 13.17%) and South Korea (313, 9.72%). USA (35,968), Sweden (9671) and Japan (9671) were cited most frequently, indicating greater international influence.

**Figure 3.**
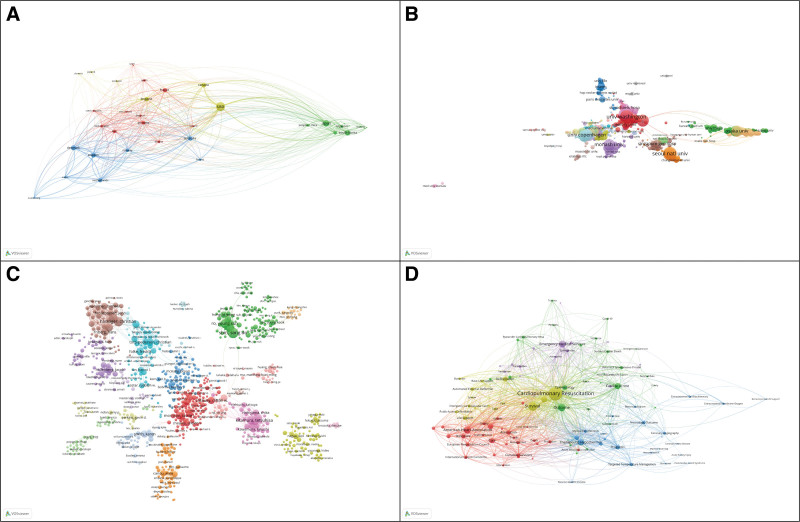
Network visualization map for cluster analysis based on international, institutions and authors collaboration, and keyword co-occurrence analysis.

Out of 120 countries, 38 countries produced at least 8 articles to identify cooperation (Fig. 3A). The size of the nodes indicates the article number, and nodes represent different countries/territories. It is indicated that there is a cooperative relationship between 2 nodes by links between them. The stronger the connection is, the greater the cooperation between the 2 countries/territories. Notably, the level of cooperation between the United States and Canada was high enough, and there was a close connection between the research communities and other clusters and countries. According to the cluster analysis, international cooperation was divided into 4 clusters. Cluster 1 included 12 countries, with France and Germany having the most publications (shown in red); Cluster 2 included 11 countries, in which Japan and South Korea were the countries with the highest number of publications (shown in green); Cluster 3 included 8 countries (shown in blue), and the top 2 countries for the number of published publications were Denmark and Sweden; Cluster 4 included 6 countries with the United States and Canada having the most (shown in yellow). These results were most likely related to geographical locations. For example, the green cluster mainly included some Asian countries. Additionally, these results may also be related to the mode of emergency medical services. For example, France and Germany belonged to Cluster 1, and the USA and Canada belonged to Cluster 4. Emergency medicine services classification evolved around 2 main models with distinct features, including the Anglo-American and Franco-German models.^[16,17]^

#### 3.2.2. Active institutions.

This bibliometric study included 3766 institutions. The top 10 research organizations with the most publications were analyzed to identify the most important research organizations in Table S1 (see Table S1, http://links.lww.com/MD/J563, Supplemental Digital Content, which illustrates the number of publications on out-of-hospital cardiac arrest of countries/territories, institutions, authors, journals, and research areas). These institutions were from the USA, South Korea, Denmark, France and Canada. The University of Copenhagen (209, 6.49%) ranked first in the number of published articles, indicating powerful production. University of Washington (11,665) was cited most frequently, indicating that it had a leading role to play in this field, and it was recognized more widely than any other organization.

The number of articles published and cited by the institution could reflect those that are the most productive and influential, as well as those that are specialized in a particular field. In Figure 3B, we depict the knowledge domain map for the coauthoring organizations. Each node represents an institution, and its size is correlated with the number of publications. As a measure of the strength of cooperation between 2 institutions, the thickness of the connecting lines was used. The majority of institutions had extensive cooperative relationships.

#### 3.2.3. Active authors.

A total of 12 083 authors were included in this study on OHCA. The 3 authors who published the most articles on OHCA were Shin SD (119, 3.70%), Hassager C (95, 2.95%) and Song KJ (95, 2.95%). Figure 3C presents a knowledge domain map for the coauthorship network of authors on OHCA. The blue and red clusters, for example, appear to have closely collaborated authors with strong subgroup connections. There was, however, a large amount of collaboration between authors who were of the same nationality and who shared the same institutional background. Although green and yellow clusters were in a marginal position, they had an influence on scientific outputs. Cross-disciplinary, interinstitutional, and cross-national collaboration as well as a role for interdisciplinary cooperation were important in the development of OHCA research, which contributed to the leap forward and diversified development of OHCA through mutual learning among various teams.

#### 3.2.4. Active journals.

In academic and scientific presentations, journals were the most important sources and indicators. Based on the results of this survey, 416 different journals published research results on OHCA. According to the search results, the 3 journals that published the most articles on OHCA were Resuscitation (952, 29.57%; IF = 6.251), American Journal of Emergency Medicine (112, 3.48%; IF = 4.093) and Circulation (77, 2.39%; IF = 39.918). The average IF 2022 of these journals was 9.091, suggesting that the quality of journals was generally high. Resuscitation (30,977), Circulation (9029) and JAMA-Journal of the American Medical Association (6925) were cited most frequently, indicating that they were more widely recognized in the OHCA field.

#### 3.2.5. Active research areas.

Due to the characteristics of OHCA, the research areas of this subject focused on medicine-related disciplines. The publications on OHCA involved many disciplines, including but not limited to Emergency Medicine (1576, 48.96%), Critical Care Medicine (1265, 39.30%) and Cardiac Cardiovascular Systems (629, 19.54%), and mainly covered medical-related subject categories (see Table S1, http://links.lww.com/MD/J563, Supplemental Digital Content, which illustrates the number of publications on out-of-hospital cardiac arrest in countries/territories, institutions, authors, journals, and research areas). Therefore, the role of interdisciplinary research should be emphasized in the development process of OHCA research.

### 3.3. Keyword co-occurrence analysis

The distribution of keywords can be used to identify the core direction and value of this field of research (see Table S1, http://links.lww.com/MD/J563, Supplemental Digital Content, which illustrates the number of publications on out-of-hospital cardiac arrest of countries/territories, institutions, authors, journals, and research areas). The 3 keywords that published the most articles on OHCA were out-of-hospital cardiac arrest (2159, 67.07%), CRP (1421, 44.14%) and survival (417, 12.95%) (see Fig. S1, http://links.lww.com/MD/J564, Supplemental Digital Content, which illustrates the high-frequency keyword cloud).

COOC of keywords in selected articles were investigated to describe the core content and structure of specific fields. We have visualized and analyzed the mapped knowledge domains of keywords in Figure 3D. A total of 335 keywords were found with a frequency >15 for cluster analysis. A total of 5 clusters were obtained. Cluster 1 (red) was mainly centered around the keyword “statement”; cluster 2 (green) was mainly centered around the keyword “epidemiology”; cluster 3 (blue) was mainly centered around the keyword “clinical care”; cluster 4 (yellow) was mainly centered around the keyword “factors influencing prognosis”; and cluster 5 (purple) was mainly centered around the keyword “emergency medical services.” Cluster 2 (Epidemiology) had a close cooperative relationship with other clusters. The potential reason was that epidemiology was a methodologic discipline.

**Figure 4.**
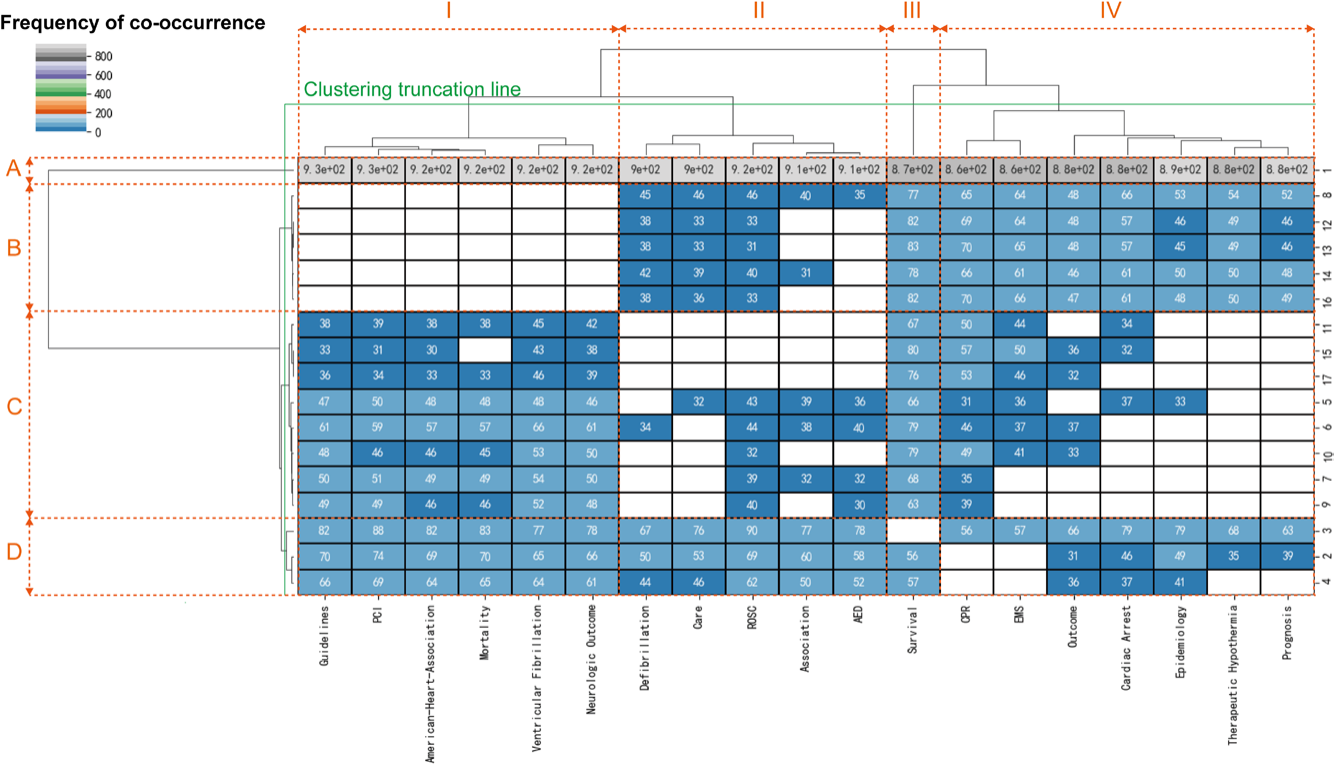
Mapping knowledge domain of system clustering pedigree tree based on a 2-module matrix of keywords and journals.

Notes: The vertical coordinates indicated the order of publication frequency. 1 Resuscitation; 2 American Journal Of Emergency Medicine; 3 Prehospital Emergency Care; 4 Circulation; 5 Critical Care; 6 Resuscitation Plus; 7 Scandinavian Journal Of Trauma Resuscitation & Emergency Medicine; 8 Plos One; 9 Journal Of The American Heart Association; 10 Journal Of Clinical Medicine; 11 Critical Care Medicine; 12 Bmj Open; 13 Emergency Medicine Journal; 14 Annals Of Emergency Medicine; 15 European Heart Journal-Acute Cardiovascular Care; 16 American Journal Of Cardiology; 17 Circulation Journal.

In the case of a hierarchical cluster analysis, it is possible to directly determine the close relationship and degree of correlation between keywords and journals.^[18]^ With a 2-mode matrix, a hierarchical cluster analysis improves the analysis of the traditional system clustering algorithm and allows simultaneous clustering of keywords and journals. Hierarchical cluster analysis mapping indicates that the closer the distance between keywords was, the greater the degree of similarity. Keywords with longer distances form different branch groups. On the right side of Figure 4, the horizontal clustering tree represents the clustering results of 21 high-frequency topic words (only showing cooccurrence frequencies of more than 70). As in the horizontal clustering tree, the serial numbers indicate the order of frequency for 17 high-frequency journals (only showing a cooccurrence frequency of more than 30) listed at the bottom of the picture. Columns in each box correspond to the high-frequency keyword-journal unit, and colors correspond to the frequency of COOC in publications (only showing COOC over 30). We identified hot research directions and popular periodical groups in the field through clustering results interpretations of high-frequency keywords and high-frequency periodicals.

The keyword hotspots concentrated on 4 aspects: (I) study on OHCA with cardiac causes, covering “Guidelines,” “PCI,” “American-Heart-Association,” “Mortality,” “Ventricular Fibrillation” and “Neurologic Outcome”; (II) influencing factors of return of spontaneous circulation (ROSC), referring to “Defibrillation,” “care,” “ROSC,” “Association,” etc; (III) research on “Survival” of OHCA; and (IV) research on the OHCA epidemiology, covering “CPR,” “EMS,” “Outcome,” “Cardiac Arrest,” “Epidemiology,” “Therapeutic Hypothermia” and “Prognosis,” etc. Categories (I) and (II) and categories (III) and (IV) could be grouped into 1 category, indicating their integration.

Accordingly, popular journals may also be classified according to unit relevance into 4 categories: (A) Resuscitation, a medical journal focused on cardiac arrest etiology, pathophysiology, and prevention; (B) Comprehensive medical journals, publishing medical research from all disciplines and therapeutic areas, such as emergency medicine and cardiovascular disease; (C) Clinical medicine and critical care journal group; and (D) Emergency Medicine journal group. Categories (C) and (D) could be further classed into 1 category, showing the exchange and integration among them focusing on clinical care. There were strong 2-dimensional connections between the (C) and (D) journal clusters and the (I) keyword cluster, indicating that the clinical medicine, critical care and emergency medicine-related articles focused on the clinical treatment of OHCA with cardiac causes. (B) Journals had significant 2-dimensional correlations with (III) and (IV) keyword clusters, indicating that more articles about influencing factors of OHCA survival and epidemiology were published in comprehensive medical journals.

### 3.4. Cocitation analysis

#### 3.4.1. Journal cocitation analysis.

Having 2 publications cited in the references of a third publication is known as a cocitation.^[19]^ We can see a map of the knowledge domains that have been mapped for journal cocitations in the field of OHCA. If a line is drawn between 2 journals, it means that 2 articles from different journals have been cited in the same article, while the thickness of the connecting line conveys the strength of cocitation between the 2 journals (see Fig. S2, http://links.lww.com/MD/J565, Supplemental Digital Content, which illustrates mapped knowledge domains for journal cocitation in the field of OHCA). The largest node was represented by Resuscitation, indicating that it was the most frequently cited journal. As a result, the number of articles published by the journal increased as well as the influence of the journal. Cluster 1 (red) mainly consisted of comprehensive and public health journals; cluster 2 (green) was mainly centered around the topic “Clinical care”; cluster 3 (blue) was mainly centered around the keyword “Critical care”; and cluster 4 (yellow) was mainly centered around the keyword “Cardiopulmonary.”

#### 3.4.2. Literature cocitation analysis.

The top 20 articles with the highest number of citations in the field of OHCA were collected and are presented in Table 1. The most frequently cited paper was “Treatment of comatose survivors of out-of-hospital cardiac arrest with induced hypothermia” published by Stephen A Bernard et al in New England Journal of Medicine.^[20]^ The study compared the effects of treatment with hypothermia or normothermia in patients who remained unconscious after resuscitation from OHCA. The second was “Regional variation in out-of-hospital cardiac arrest incidence and outcome” by Graham Nichol et al (2008) in JAMA-Journal of the American Medical Association. As a comprehensive epidemiological investigation, the study reported OHCA incidence and outcome in 10 North American sites.^[21]^ For subjects, 8 of the top twenty publications investigated the treatment of OHCA, of which 3 publications focused on CPR, and others focused on amiodarone, percutaneous coronary intervention and TH. There were 6 and 3 articles in epidemiology research and research about factors influencing outcomes, respectively. Moreover, 3 articles on the application of initiatives, standardized treatment protocols and registry templates also deserved our attention. These articles focused on 2 key questions: “What is the current epidemic status of OHCA?” and “How can the survival rate of OHCA be improved?”

**Table 1:** Top 20 publications with the most citations in the field of out-of-hospital cardiac arrest.

Rank	Title	Journal	Authors	Yr	Citations	IF
1	Treatment of comatose survivors of out-of-hospital cardiac arrest with induced hypothermia	New England Journal of Medicine	Bernard SA, et al	2002	3906	176.079
2	Regional variation in out-of-hospital cardiac arrest incidence and outcome	Jama-Journal of the American Medical Association	Nichol G, et al	2008	1492	157.335
3	Predictors of survival from out-of-hospital cardiac arrest a systematic review and meta-analysis	Scientific Research Forum of the American-College-Of-Emergency-Physicians	Sasson C, et al	2010	1400	/
4	Global incidences of out-of-hospital cardiac arrest and survival rates: systematic review of 67 prospective studies	Resuscitation	Berdowski J, et al	2010	1217	6.251
5	Quality of cardiopulmonary resuscitation during out-of-hospital cardiac arrest	Jama-Journal of the American Medical Association	Wik L, et al	2005	999	157.335
6	Public-access defibrillation and survival after out-of-hospital cardiac arrest	New England Journal of Medicine	Hallstrom AP, et al	2004	802	176.079
7	Association of national initiatives to improve cardiac arrest management with rates of bystander intervention and patient survival after out-of-hospital cardiac arrest	Jama-Journal of the American Medical Association	Wissenberg M, et al	2013	768	157.335
8	Implementation of a standardized treatment protocol for post resuscitation care after out-of-hospital cardiac arrest	Resuscitation	Sunde K, et al	2007	684	6.251
9	Advanced cardiac life support in out-of-hospital cardiac arrest	New England Journal of Medicine	Stiell IG, et al	2004	627	176.079
10	Amiodarone for resuscitation after out-of-hospital cardiac arrest due to ventricular fibrillation	New England Journal of Medicine	Kudenchuk PJ, et al	1999	623	176.079
11	Cardiac arrest and cardiopulmonary resuscitation outcome reports: update of the utstein resuscitation registry templates for out-of-hospital cardiac arrest a statement for healthcare professionals from a task force of the international liaison committee on resuscitation	Circulation	Perkins GD, et al	2015	607	39.918
12	Incidence of ems-treated out-of-hospital cardiac arrest in Europe	Resuscitation	Atwood C, et al	2005	599	6.251
13	Immediate percutaneous coronary intervention is associated with better survival after out-of-hospital cardiac arrest insights from the procat (Parisian region out of hospital cardiac arrest) registry	Circulation-Cardiovascular Interventions	Dumas F, et al	2010	533	7.514
14	Early cardiopulmonary resuscitation in out-of-hospital cardiac arrest	New England Journal of Medicine	Hasselqvist AI, et al	2015	519	176.079
15	Eureca 1-27 nations, 1 Europe, 1 registry a prospective 1 mo analysis of out-of-hospital cardiac arrest outcomes in 27 countries in Europe	Resuscitation	Grasner JT, et al	2016	496	6.251
16	Epidemiology and outcomes from out-of-hospital cardiac arrest in children the resuscitation outcomes consortium epistry-cardiac arrest	Circulation	Atkins DL, et al	2009	480	39.918
17	Reversible myocardial dysfunction in survivors of out-of-hospital cardiac arrest	Journal of the American College of Cardiology	Laurent I, et al	2002	466	27.203
18	Minimally interrupted cardiac resuscitation by emergency medical services for out-of-hospital cardiac arrest	Jama-Journal of the American Medical Association	Bobrow BJ, et al	2008	400	768
19	Recent trends in survival from out-of-hospital cardiac arrest in the United States	Circulation	Chan PS, et al	2014	380	39.918
20	Incidence of ems-treated out-of-hospital cardiac arrest in the United States	Resuscitation	Rea TD, et al	2004	371	6.251

IF = impact factor.

#### 3.4.3. Coupled analysis.

The concept of coupled papers was based on the assumption that the more similar articles were to each other, the more of the same articles they contained in their references. When 2 papers cited one another, the 2 papers were called coupled papers. Cocitation network clustering differed from bibliographic coupling network clustering. Cocitation matrix based on the former, coupling matrix based on the latter. The coupled network graph may be useful in analyzing the contributions and contacts of authors in the field and identifying potential collaborators. Knowledge domains for coupled journals in OHCA are mapped (see Fig. S3A, http://links.lww.com/MD/J566, Supplemental Digital Content, which illustrates mapped knowledge domains for coupled journals in the field of OHCA). If 2 articles from different journals cited 1 article, the thickness of the connecting line indicates the degree of coupling between them. The largest node represented resuscitation. Cluster 1 (red) was mainly centered around the topic “Cardiopulmonary”; cluster 2 (green) was mainly centered around the topic “Clinical Care”; and cluster 3 (blue) was mainly centered around the keyword “Critical care.” According to the cluster analysis, 5 different clusters related to coupled authors were formed in the field of OHCA, suggesting potential cooperative partners in future research (see Fig. S3B, http://links.lww.com/MD/J566, Supplemental Digital Content, which illustrates mapped knowledge domains for coupled authors in the field of OHCA).

### 3.5. Frontier identification

#### 3.5.1. Timeline analysis.

**Figure 5.**
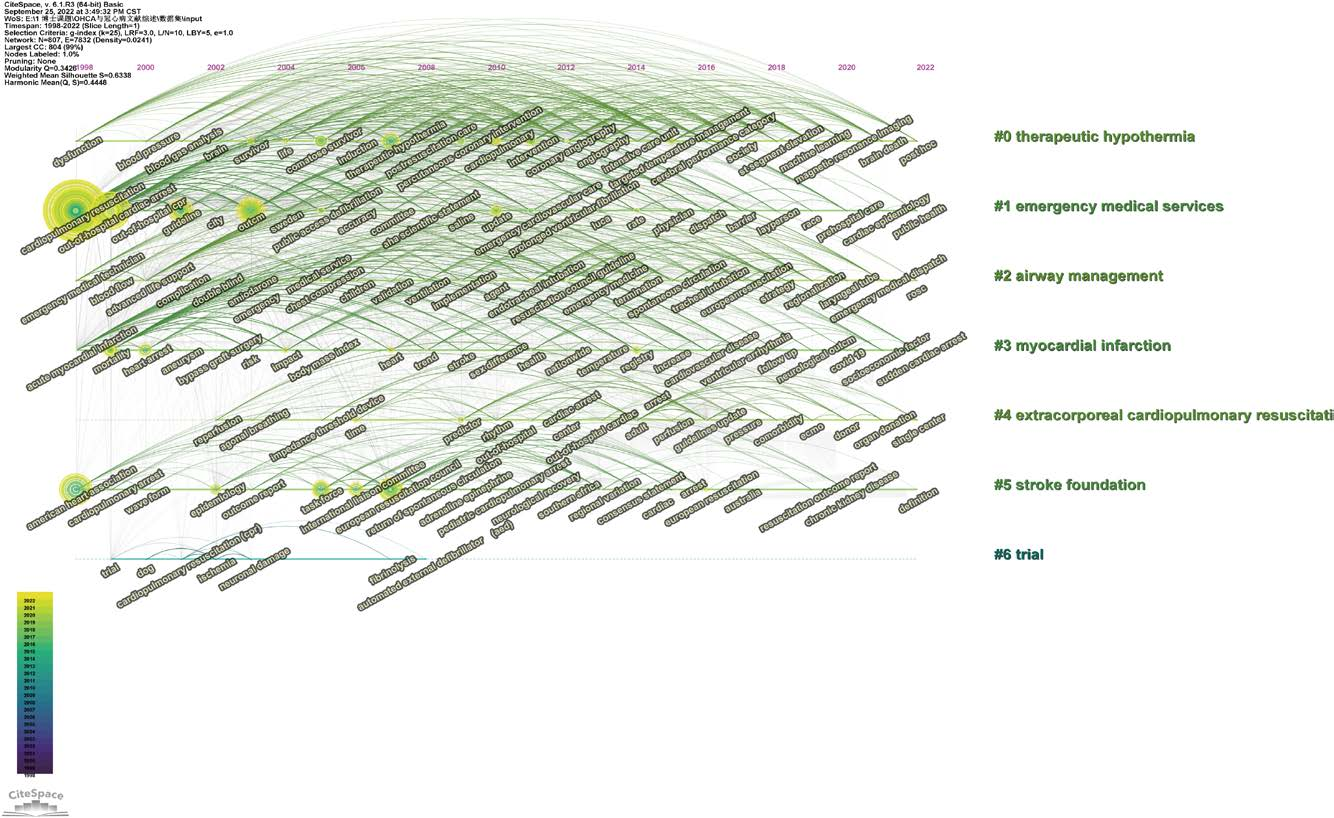
Timeline view of keywords and the cluster labels.

Furthermore, this study explored the frontier hotspots and evolution of OHCA research from 1998 to 2022 through keyword COOC analysis. Clustering keywords over time were evaluated based on the relationship and interaction between clusters, as well as their historical span and evolution. There are horizontal and vertical axes in the timeline view, which represent the time at which the node arose as well as the cluster to which it belonged, respectively. According to the occurrence time sequence, the nodes of the same cluster were arranged on the same horizontal line based on the order in which they occurred. As nodes grew, they burst stronger, and connecting lines showed cooccurrence relationships. There are 7 categories of keywords, with several keywords in each category (Fig. 5).

Cluster #0: therapeutic hypothermia (TH). In the aftermath of OHCA, TH has been shown to have the potential to improve survival and neurological outcomes in patients.^[22]^ A study conducted by the National Institutes of Health found that cooling the body to 32 to 34°C resulted in a 35% increase in survival rate compared with no treatment.^[23]^ A recent study suggests that cooling to 36°C has similar health benefits to cooling to 32 to 34°C, according to recent research.^[20]^ However, the optimal strategy, efficacy and safety of TH use remain limited.^[24,25]^ In addition, “TH” could also be identified from the top 25 keywords with the strongest citation burst from 2012 to 2016.

Cluster #1: Emergency medical services. The survival of OHCA patients depends on a coordinated set of actions (survival chain), including early recognition of cardiac arrest and activation of the emergency response system, early CPR, rapid defibrillation, effective advanced life support, and integrated care following cardiac arrest.^[26]^ As part of the chain of survival, there is the community, emergency medical services, ambulance services, and hospital-based services.^[27]^ In the past few decades, there has been an increasing awareness of the importance of emergency medical services, the community involvement, and the role of emergency medical dispatch centers in providing these services.^[5]^

Cluster #2: Airway management. In survivors of OHCA, optimal CRP and ROSC are critical to preventing or minimizing neurological impairment.^[28]^ It appears that there is some controversy regarding the best approach to managing airway obstructions in OHCA. It has been observed that when basic airways are compared to advanced airways, there are conflicting results.^[29–31]^

Cluster #3: Myocardial infarction. The causes of OHCA can be broadly categorized into cardiac and noncardiac causes.^[32]^ Investigators found coronary thrombus in 74 of 100 patients who died from sudden cardiac ischemia.^[33]^ Acute myocardial infarction is the main recognized cause of OHCA.^[1]^ Left ventricular ejection fraction has been shown to have limited effectiveness as a risk stratification tool for OHCA due to cardiac causes, and the field has since shifted its focus from the “high-risk ejection fraction” to the broader concept of the “high-risk patient.” In addition to sociodemographic characteristics and lifestyle, previous medical histories, physical examinations, and laboratory results, researchers are studying possible OHCA risk factors.^[34,35]^

Cluster #4: Extracorporeal cardiopulmonary resuscitation (ECRP). ECPR can be a potentially beneficial option for patients who suffer from OHCA.^[36]^ There have been several previous observational studies regarding the treatment of OHCA with ECPR that have shown favorable survival rates and an encouraging neurological outcome when strict inclusion and exclusion criteria are applied to distinct patient populations with OHCA.^[37,38]^ Most studies have been conducted at a single center, but multicenter studies have been published in recent years.^[39,40]^ From the top 25 keywords with the strongest citation burst in 2022, “extracorporeal cardiopulmonary resuscitation” can be identified.

Cluster #5: Stroke foundation. The Stroke Foundation participated in the development of some related templates and guidelines.^[41]^ Stroke is one of the causes of OHCA.^[42]^ Studies have shown that target temperature management or TH is an effective neuroprotective strategy after cardiac arrest and after stroke.^[43]^

Cluster #6: Trial. Multiple animal and human trials using drugs, devices and treatment procedures have been performed in an attempt to improve survival in OHCA patients.^[44,45]^

#### 3.5.2. Burst detection analysis.

**Figure 6.**
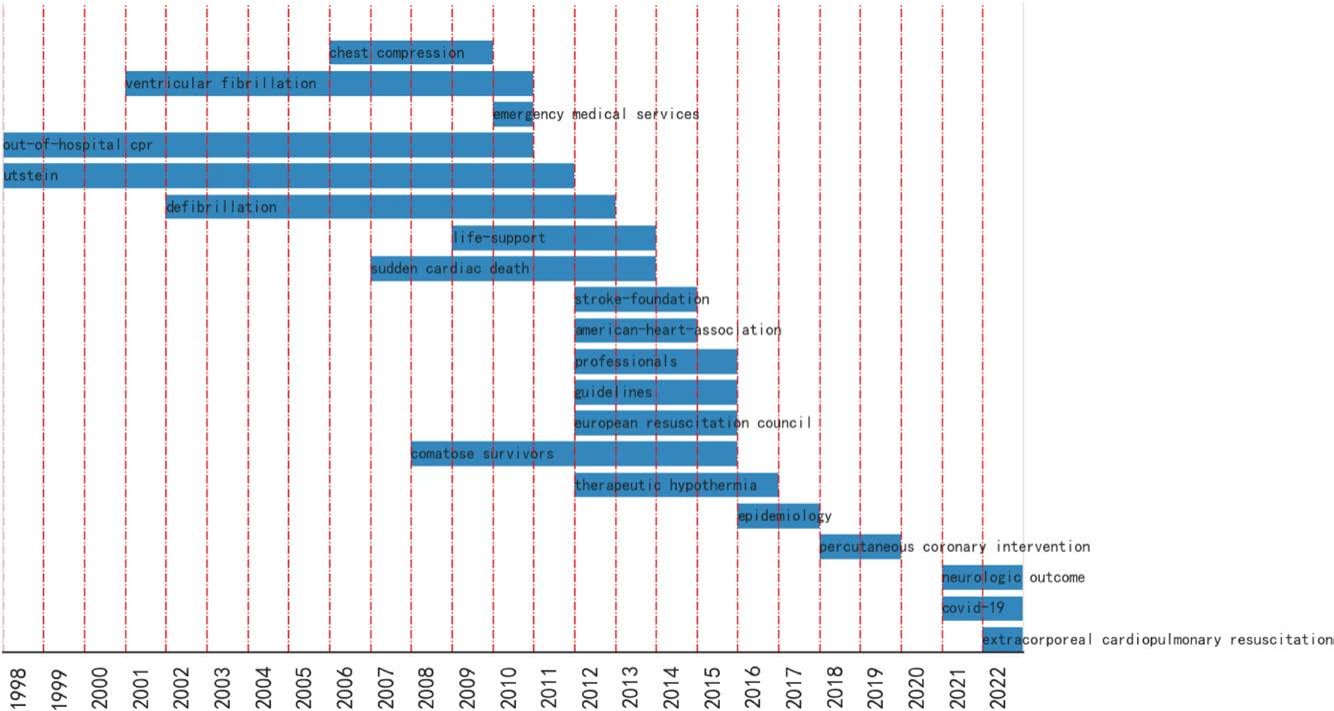
Top 25 keywords with the most burst strength.

Burst detection analysis is an essential tool for identifying research hotspots and development trends in the research field. The keywords of burst were “chest compression,” “ventricular fibrillation,” “out-of-hospital CRP,” “ustein,” “defibrillation,” “sudden cardiac death” and “comatose survivors” in the initial period, and these generally had relatively long burst durations (Fig. 6). In addition, the burst keywords included “life-support,” “stroke-foundation,” “American-heart-association,” “professionals,” “guidelines,” “European resuscitation council,” “TH,” “epidemiology,” “percutaneous coronary intervention,” “neurologic outcome,” “COVID-19” and “extracorporeal cardiopulmonary resuscitation” in the development period. Thirteen of the top twenty burst keywords were published after 2008, indicating that the field developed rapidly during this period of time. In the last 5 years, the burst words were “percutaneous coronary intervention,” “neurologic outcome,” “COVID-19” and “extracorporeal cardiopulmonary resuscitation,” indicating that they were the most emerging topics. The keywords with the longest burst period were “out-of-hospital CPR” and “Utstein,” which had a more profound influence on this research field. When CPR is started in OHCA at an early stage, the chances of survival are higher. A key modifiable factor for survival after OHCA was out-of-hospital CPR. A number of published studies of cardiac arrest have used Utstein-style definitions and reporting templates, leading to a better understanding of resuscitation elements for uniformity in defining and reporting results and progress toward international consensus on science and resuscitation guidelines.^[41]^

### 3.6. Limitations

This study also has some limitations. First, our analyses only used the WoS core collection database. We took into account the features of the WoS core collection database, such as having a broad network of journals, already having numerous important studies from other databases, and providing enough data for citation and cocitation analyses.^[46]^ Most of the bibliographic studies used the WoS core collection database.^[47,48]^ The second problem is that there are still some problems with the visualization, such as cluster overlap and a lack of flexibility in the setting of parameters. Third, to avoid the occurrence of less relevant or even completely irrelevant references to the paper, it is urgent that a new approach is taken to quantify the relevance between the references cited and the article to avoid the occurrence of less relevant or even completely irrelevant references. A general trend in the future will be to establish and unify authoritative standards and method systems in bibliometrics, a newly developing field.

## 4. Conclusions

Herein, a bibliometric analysis was performed based on 3219 OHCA-related publications from 1998 to 2022. The main important research findings obtained are as follows:

1. The overall trend could be divided into initial budding (before 2008) and rapid development (after 2008). The USA was the most active country, and the University of Copenhagen and Resuscitation were the most active institutions and journals respectively. There is relatively close global cooperation at present. The research areas of this subject focused on medicine-related disciplines, and the role of interdisciplinary research should be emphasized.
2. In addition to OHCA, the most frequent keywords were Cardiopulmonary Resuscitation, Survival, Emergency Medical Services and Outcome. Five research hotspots were identified: statement, epidemiology, clinical care, factors influencing prognosis and emergency medical services. The research on OHCA had the most frequent interaction with OHCA with cardiac causes, influencing factors of ROSC, OHCA survival and OHCA epidemiology research.
3. Resuscitation was the most recognized authoritative communication platform. Topics such as treatment, epidemiology, factors influencing outcome, the application of initiatives, standardized treatment protocol and registry templates of OHCA were the research hot spots in the most cited article analysis.
4. Seven categories were classified via the bibliometric method, including TH, emergency medical services, airway management, myocardial infarction, extracorporeal cardiopulmonary resuscitation, stroke foundation and trial. These articles focused on 2 key questions: “What is the current epidemic status of OHCA?” and “How can the survival rate of OHCA be improved?,” which would be the frontier issues in the future.

## Acknowledgments

Thank Xueshudiandi Team for the developing of COOC 12.8 software, which is made available for use free of charge.

## Author contributions

**Conceptualization:** Yue Li.

**Funding acquisition:** Haojun Fan, Chunxia Cao.

**Methodology:** Yue Li, Zhaoying Li, Tao Liu.

**Software:** Wei Cai, Tao Liu, Ji Li.

**Visualization:** Wei Cai, Ji Li.

**Writing – original draft:** Yue Li, Zhaoying Li, Chunjie Li.

**Writing – review & editing:** Haojun Fan, Chunxia Cao.

## Supplementary Material










