# Supplementary material for: Out-of-hospital cardiac arrest: A data-driven visualization of collaboration, frontier identification, and future trends
Source: Medicine (Baltimore). 2023 Aug 18;102(33):e34783. doi: 10.1097/MD.0000000000034783 (PMC10443760; doi:10.1097/MD.0000000000034783)
Supplement: Supplementary file 1 [file medi-102-e34783-s001.pdf]

**The diagram of the construction about two-mode matrix relation**

1) Items extracted from the original data

| Publications | J items | K items |
|---|---|---|
| 1 | j1, j2 | k1, k2 |
| 2 | j2, j3 | k1, k2, k3 |
| 3 | j1, j3 | k3, k4 |
| 4 | j1, j3, j4 | k3 |

2) Two-mode matrix table generated by counting and statistics

| Items | k1 | k2 | K3 | K4 |
|---|---|---|---|---|
| j1 | 1 | 1 | 2 | 1 |
| j2 | 1 | 1 | 1 | 0 |
| j3 | 0 | 0 | 2 | 1 |
| j4 | 0 | 0 | 1 | 0 |

For example:

□. If the J/K item in 1) are journals/keywords, the result is the Journals-Keywords two-mode matrix.

□. If the one items in 1) are citations, the result is the citation coupling matrix used for bibliographic coupling analysis.

Supplement:

1. After the matrix is constructed, the data are imported into the drawing software (COOC/VOSviewer) for visualization.

2. In the clustering analysis, the distance algorithm between samples is Euclidean distance algorithm, the clustering method is the Ward’s minimum-variance method, and the matrix standardization method uses Z-Sore standardization.
