# Supplementary material for: Out-of-hospital cardiac arrest: A data-driven visualization of collaboration, frontier identification, and future trends
Source: Medicine (Baltimore). 2023 Aug 18;102(33):e34783. doi: 10.1097/MD.0000000000034783 (PMC10443760; doi:10.1097/MD.0000000000034783)
Supplement: Supplementary file 2 [file medi-102-e34783-s002.pdf]

Table S1 Listing of the number of publications on out-of-hospital cardiac arrest of countries/territories, institutions, authors, journals, research areas

Variable	Number of Publications, n (%)
**Countries/territories**	
USA	824 (25.60%)
Japan	424 (13.17%)
South Korea	313 (9.72%)
Denmark	258 (8.01%)
Sweden	249 (7.74%)
Canada	240 (7.46%)
China	226 (7.02%)
England	203 (6.31%)
Australia	202 (6.28%)
France	195 (6.06%)
**Institutions**	
University of Copenhagen	209 (6.49%)
Seoul National University Snu	169 (5.25%)
Rigshospitalet	152 (4.72%)
Udice French Research Universities	148 (4.60%)
University of Washington	148 (4.60%)
University of Washington Seattle	148 (4.60%)
University of Toronto	133 (4.13%)
Pennsylvania Commonwealth System of Higher Education Pcshe	131 (4.07%)
Seoul National University Hospital	128 (3.98%)
University of Pittsburgh	127 (3.95%)
**Authors**	
Shin SD	119 (3.70%)
Hassager C	95 (2.95%)
Song KJ	95 (2.95%)

Iwami T	94 (2.92%)
Kitamura T	93 (2.89%)
Herlitz J	90 (2.80%)
Kjaergaard J	84 (2.61%)
Ro YS	78 (2.42%)
Nielsen N	72 (2.24%)
Friberg H	67 (2.08%)
**Journals**	
Resuscitation	952 (29.57%)
American Journal of Emergency Medicine	112 (3.48%)
Circulation	77 (2.39%)
Prehospital Emergency Care	77 (2.39%)
Critical Care	70 (2.17%)
Resuscitation Plus	61 (1.89%)
Scandinavian Journal of Trauma Resuscitation Emergency Medicine	59 (1.83%)
Journal of the American Heart Association	51 (1.58%)
Plos One	51 (1.58%)
Journal of Clinical Medicine	48 (1.49%)
**Research areas**	
Emergency Medicine	1576 (48.96%)
Critical Care Medicine	1265 (39.30%)
Cardiac Cardiovascular Systems	629 (19.54%)
Medicine General Internal	380 (11.80%)
Public Environmental Occupational Health	140 (4.35%)
Peripheral Vascular Disease	108 (3.36%)
Multidisciplinary Sciences	84 (2.61%)
Anesthesiology	65 (2.02%)
Medicine Research Experimental	38 (1.18%)
Health Care Sciences Services	30 (0.93%)

**Keywords**	
Out Of Hospital Cardiac Arrest	2159 (67.07%)
Cardiopulmonary Resuscitation	1421 (44.14%)
Survival	417 (12.95%)
Emergency Medical Services	368 (11.43%)
Outcome	343 (10.66%)
Cardiac Arrest	296 (9.20%)
Therapeutic Hypothermia	273 (8.48%)
Ventricular Fibrillation	193 (6.00%)
Prognosis	189 (5.87%)
Epidemiology	154 (4.78%)
