# Supplementary material for: Out-of-hospital cardiac arrest: A data-driven visualization of collaboration, frontier identification, and future trends
Source: Medicine (Baltimore). 2023 Aug 18;102(33):e34783. doi: 10.1097/MD.0000000000034783 (PMC10443760; doi:10.1097/MD.0000000000034783)

Supplemental Digital Content. Figure S2 illustrates mapped knowledge domains for journal cocitation in the field of OHCA

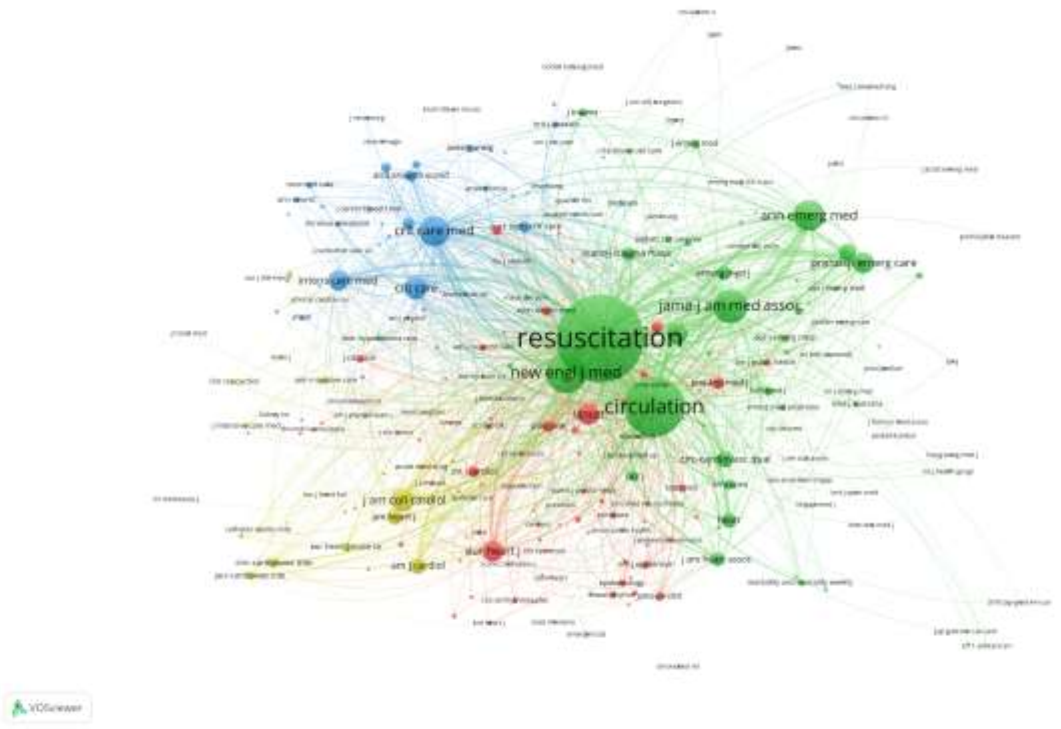

Supplement: Supplementary file 4 [file medi-102-e34783-s004.pdf]
